# Bacterial meningitis in Sudanese children; critical evaluation of the clinical decision using clinical prediction rules

**DOI:** 10.1186/s12887-019-1684-3

**Published:** 2019-09-06

**Authors:** Nada Abdelghani Abdelrahim, Imad Mohammed Fadl-Elmula, Hassan Mohammed Ali

**Affiliations:** 1 Department of Pharmaceutics-Medical Microbiology, Faculty of Pharmacy, Nile University, Hai El-Gamaa, Al-Ailafoon Road, East Manshya Bridge, P.O. Box 11111, Khartoum, Sudan; 2 Department of Pathology & Clinical Genetics, Alneelain University & Assafa Academy, Khartoum, Sudan; 3 Department of Clinical Pharmacology, Faculty of Pharmacy, National University-Sudan, Khartoum, Sudan

**Keywords:** Bacterial meningitis, Bacterial meningitis score, Children, Sudan

## Abstract

**Background:**

Sudan falls in the meningitis belt where most global cases of bacterial meningitis are reported. Highly accurate decision support tools have been developed by international specialized societies to guide the diagnosis and limit unnecessary hospital admissions and prolonged antibiotic use that have been frequently reported from countries around the world. The goals of this study are to critically evaluate the clinical decision of bacterial meningitis in children in Sudan using clinical prediction rules and to identify the current bacterial aetiology.

**Methods:**

This cross-sectional hospital-based study was conducted in October to July of 2010 in a major referral pediatric hospital in Khartoum, Sudan. Febrile children age 1 day to 15 years who were provisionally diagnosed as having meningitis on admission were included (*n* = 503). Cerebrospinal fluid (CSF) specimens were obtained from all patients while clinical and demographic data were available for only 404. Conventional laboratory investigations were performed. The clinical decision was evaluated by the International Classification of Diseases–Clinical Modification code 320.9 and the Bacterial Meningitis Score. Ethical clearance and permissions were obtained.

**Results:**

Out of 503 provisionally diagnosed bacterial meningitis patients, the final clinical confirmation was assigned to 55.9%. When codes were applied; 5.7% (23/404) with CSF pleocytosis were re-classified as *High Risk* for bacterial meningitis and 1.5% (6/404) with confirmed bacterial aetiology as *Proven Bacterial Meningitis. Neisseria meningitidis* was identified in 0.7% (3/404) and *Streptococcus pneumoniae* in another 0.7%. Typical laboratory findings (i.e. CSF pleocytosis and/or low glucose and high protein concentrations, Gram positive or Gram negative diplococcic, positive bacterial culture) were seen in 5 (83%). Clinically, patients showed fever, seizures, chills, headache, vomiting, stiff neck and bulging fontanelle. All confirmed cases were less than 5 years old and were admitted in summer. All patients were prescribed with antibiotics; they were all recovered and discharged.

**Conclusions:**

Bacterial meningitis is over-diagnosed in hospitals in Khartoum therefore clinical prediction rules must be adopted and applied to guide the clinical decision. The sole bacterial aetiology in this selected group of Sudanese children remain *N. meningitidis* and *S. pneumoniae*, but with significant decrease in prevalence. Some cases showed atypical clinical and laboratory findings.

**Electronic supplementary material:**

The online version of this article (10.1186/s12887-019-1684-3) contains supplementary material, which is available to authorized users.

## Background

Bacterial meningitis (BM) can be a life-threatening emergency if not properly diagnosed and managed [1]. Over 1.2 million cases of BM are estimated to occur worldwide annually [2]. Incidence and case-fatality rates vary by region, country, pathogen and age group [3]. Case-fatality rate can be as high as 70% in untreated patients and 1 in 5 survivors may be left with permanent sequelae [3]. Therefore, BM is considered as one of the most feared childhood diseases. Consequently, the WHO developed recommendations for detecting BM epidemics in highly endemic countries in Africa (*Alert* and *Epidemic* definitions) [4, 5].

The epidemiology of BM has changed dramatically over the last 20 years, primarily as a result of the introduction of conjugate vaccines [1] against the commonest meningeal pathogens; *Neisseria meningitidis*, *Streptococcus pneumoniae* and *Haemophilus influenzae* [2]. Worldwide, the incidence of meningitis due to *N. meningitidis* is highest in the meningitis belt; a region of sub-Saharan Africa described as hyper-endemic and epidemics occurring during the dry season (December to June) [5, 6]. Statistically, the incidence rate is 10 to 100 cases per 10^5^ populations punctuated by explosive epidemics in 8 to 12 year cycles with incidence rates that can be greater than 10^3^ cases per 10^5^ populations [5, 6]. Across the meningitis belt, at least 350 million people are at risk for meningitis during these annual epidemics [6]. The climato-geographic location renders Sudan at permanent risk. A total of 15,595 cases including 1670 deaths due to *N. meningitidis* were reported from 4 countries in the meningitis belt in 2007 [7]. One of these countries was Sudan where 6946 cases with 430 deaths were reported from 9 out of 10 Southern states in the former Republic of Sudan [7]. About 1.1 million meningococcal vaccine doses were released in response to the outbreak in South Sudan targeting people in the affected areas in mass vaccination campaigns [7]. Mass vaccinations can lead to herd immunity resulting in dramatic reduction in infection rates among populations at risk [8]. The massive return of displaced Southerners to their homelands after the separation; where most areas fall in the meningitis belt, should have definitely affected the intensity and distribution of meningitis in the North; where minimum areas are considered to be within the doomed region.

Meningitis due to *S. pneumoniae* occurs most commonly in the very young and the very old, with an estimated incidence rate of 17 cases per 10^5^ in children less than 5 years, and case fatality rates that exceed 73% in some areas [9]. *H. influenzae* type b (Hib) is a major cause of infant and childhood meningitis [10]. Rates are highest in children less than 5 years reaching 31 cases per 10^5^ populations [10]. In young children, the case-fatality rate of Hib meningitis is generally higher than that for meningococcal meningitis [10]. Because of vaccination [11, 12] the burden of Hib meningitis is dramatically decreased in most industrialized countries and has been virtually eliminated as a public health problem [13]. We anticipate a comparable situation since Sudan has introduced Hib vaccines earlier than most developing countries and has maintained immunization coverage of 93% [13].

Distinguishing BM is often difficult [14] therefore several highly accurate decision support tools have been developed and validated to guide decision making and limit unnecessary hospital admissions and prolonged antibiotic use [14–16]. In Sudan, as in many other countries around the world, children who are suspected and provisionally diagnosed with meningitis are routinely admitted to hospitals and administered broad-spectrum antibiotics irrespective of culture and laboratory confirmation. Proper diagnosis of infectious CNS syndromes and the subsequent ability to distinguish BM is vital. This study aims at critically evaluating the hospital diagnosis of BM using internationally validated clinical prediction rules. We also aimed at studying the clinical parameters and identifying the type and frequency of bacterial aetiologies.

## Methods

### Study design and ethical considerations

This cross-sectional hospital-based study was conducted at a large central pediatric reference hospital in Khartoum, Sudan, during 10 months period (October to July, 2010). All febrile (>37 °C) attendees age 1 day to 15 years who were suspected of meningitis during the study period were included. Independent and dependent variables (demographic, clinical and conventional laboratory data and final outcome) were collected simultaneously in a pre-designed structured data sheet and kept anonymous. Ethical clearance was obtained from the Ethical Committee Board of Al-Neelain University. Permission to collect data was granted from hospital authorities. Patients were not contacted directly; data were obtained from hospital files.

### Study population and specimens

Hospital case definition for suspected meningitis is: “sudden onset of fever, headache, stiff neck, episodes of seizure before or during admission and/or other symptoms as; nausea, vomiting, photophobia, altered mental status and coma”. In newborns and young children: “General signs of being unwell as irritability, vomiting, poor feeding and/or bulging fontanelle”. A confirmed case is: “a clinically compatible case that is laboratory confirmed by Gram stain and culture”.

History of the current illness was evaluated upon admission by pediatricians and/or house-officers. Patients were inquired about self-medication with antibiotics 1 week prior to lumbar punctures (LP) and contact with individuals with similar illness. Patients were examined clinically to evaluate onset of the classic symptoms: fever, headache, neck-stiffness, vomiting, bulging fontanelle, chills, seizures, altered mental status, skin rash, petechiae and coma. Demographic data included age and sex. Information recorded during hospital stay were: types of antibiotics administered, duration of hospital stay, final diagnosis and outcome. Total volumes of 3 ml CSF were obtained from each patient via lumbar or ventricular puncture by hospital medical staff.

### Laboratory investigations

Color and turbidity of CSF specimens were macroscopically inspected immediately upon withdrawal. Microscopical examination was performed on wet preparations and on Gram stained smears from the sediment. White cell count was performed in the non-centrifuged portions of specimens (diluted with an isotonic 0.1% toluidine blue, 1 in 2) using modified Fuchs-Rosenthal ruled counting chamber. CSF cell count was reported as cells/mm^3^. When no white cells were seen, the count was reported as <5 cells/mm^3^. Percentages of polymorphs and lymphocytes were estimated.

GlucoseOxidase/Peroxidase® and Biuret® methods were used to measure CSF glucose level and CSF total proteins immediately upon collection using manual colorimetric methods and reagents (Biosystems®) following the supplier’s protocols.

Irrespective of Gram stain result, all CSF specimens were cultured on Blood, Chocolate and MacConkey agar media. Inoculated cultures were incubated aerobically at 35 °C overnight. Blood and Chocolate agar cultures were incubated in CO_2_ enriched atmosphere and kept for 72 h. Bacterial growth was examined daily and suitable biological and serological tests were applied to identify the aetiologic agent following the recommendations of Gray & Fedorko [17] and Vandepitte et al [18].

### Secondary analysis using published clinical prediction rules

Data collected throughout this study were used to distinguish BM according to the criteria listed in the *Bacterial Meningitis Score* (BMS) [14] which was later validated in the era of widespread pneumococcal vaccination [16] and the *International Classification of Diseases–Clinical Modification Code 320.9* (ICD-CMC320.9) for BM [15]. These criteria aim at identifying infectious meningitis and distinguishing the bacterial condition in children with CSF pleocytosis. Following the criteria, meningitis suspected cases were re-classified as BM based primarily on CSF pleocytosis and the presence of at least one of the following indicators; seizures on or before presentation, other clinical signs of meningeal irritation, CSF absolute neutrophil count, CSF Gram stain and CSF protein concentration. Cases showing CSF pleocytosis were considered as *Infectious Meningitis*, those with one or more criteria of the BMS were considered as *High Risk* for BM, cases with in vitro culture confirmed bacterial aetiology were considered as *Proven Bacterial Meningitis* which was further specified as *Meningococcal Meningitis* when the isolated bacteria was *Neisseria meningitidis* or as *Streptococcal Meningitis* when it was *Streptococcus pneumoniae*.

### Statistical analysis

The statistical package program SPSS version 21 was used. All numerical variables were organized into categories for better interpretation. Age obtained in days was converted into groups of neonates (1 to 29 days), infants (1 to 11 months), toddlers (1 to 5 years), children (6 to 10 years) and teenagers (11 to 15 years). All categorical variables were expressed in frequencies and percentages; high statistical significance (*p* < 0.001) for frequencies distribution was detected for each variable using the *General Trend Analysis*. Numerical variables were described using measures of central tendency and of dispersion. When *Shapiro-Wilk Test of Normality* was conducted, all data were found to be non-normally distributed; accordingly, nonparametric statistical tests were performed. Comparisons between categorical variables were done using cross-tabulations. Inferential statistics for statistically significant differences under the 0.05 level was applied using either *Pearson Chi-square test*; when 0 cells in the contingency table have expected count < 5, or *Fisher’s Exact test*; when ≥1 cells in the table have expected count < 5. *Nominal by Nominal Phi and Cramer’s V Correlation* (*Corre*) along with its 95% confidence intervals (CI) were obtained to assess possible relationships between categorical variables.

## Results

### Demographic data

**Fig. 1:**
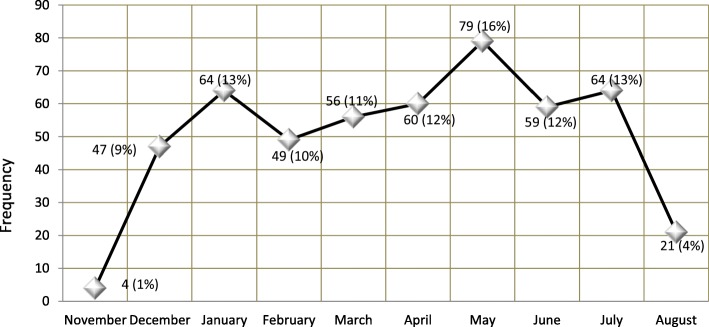
Frequency of admissions to Omdurman Hospital for Children with suspicion of meningitis (Percentages were obtained out of 502 [1 case is missing])

A total of 503 patients were included. Patients arrived from different areas surrounding the hospital in Omdurman city and were all of low socio-economic class. Most admissions were in summer; a period extending from March to June (Fig. 1). Males represent 58.4% (294/503). The median age of all children is 1.3 years with a range of 2 days to 15 years (i.e. 0.01–15.0 years). Median age among males is 1.25 years (IQR = 1.73; 0.01–15.0, *n* = 294) and among females is 1.33 years (IQR = 1.75; 0.02–14.0, *n* = 209). The most frequent age group was (1 to 5 years) among males (164/288 [6 missing], 56.9%) and among females (125/208 [1 missing], 60.1%), followed by age group (1 to 11 months) among males (104/288, 36.1%) and among females (76/208, 36.5%).

### Clinical data

The vast majority of cases with full records on clinical data (*n* = 361; 28% [142 missing]) presented with fever and seizures, other symptoms are shown on (Table 1). All patients were never in close contact with family members or relatives with similar illness, they did not receive any type of antibiotics before hospital admission. Statistically significant differences but weak associations were obtained between fever and vomiting (*p* = 0.049, *Corre* = 0.13 [95% CI: 0.03 to 0.23]). All 357 (71% out of total 503) cases, with available hospital-stay data, did not suffer from complications and were discharged.

**Table 1:** Clinical data among patients with suspected meningitis and have available clinical data (*n* = 361)

Signs & Symptoms		Number of Cases	% Out of 361
1	Fever	355	98.3%
2	Seizures	334	92.5%
3	Vomiting	189	52.4%
4	Chills	63	17.5%
5	Altered Mental Status	36	10.0%
6	Stiff neck	34	9.4%
7	Coma	11	3.0%
8	Skin Rash	3	0.8%
9	Petechiae	2	0.6%
Signs & Symptoms		Number of Cases	% Out of 309
10	Headache (for > 1 year olds)	23	7.4%
Signs & Symptoms		Number of Cases	% Out of 186
11	Bulging Fontanelle (for ≤18 months olds)	37	20%

Statistically significant difference and association were obtained between age groups and headache (*p*<0.001, *Corre* = 0.4 [95% CI: 0.30 to 0.49]). Headache, naturally, was evaluated in 1 year olds and above where most patients (65%, 15/23) were in age group (1–5 years), 13% (3/23) in age group (6–10 years) and 22% (5/23) in age group (≥11 years). Age groups and seizures showed significant difference (*p* = 0.046) but no association. Seizures were seen in all 4 patients in age group (1–29 days) and all 6 patients in (6–10 years), in 92.5% (124/134) in age group (1–11 months), 93.3% (194/208) in age group (1–5 years) and 66.7% (6/9) in age group (≥11 years). Highly significant difference was observed between season and stiff neck (*p* = 0.00) accompanied with significant but weak association (*Corre* = 0.23 [95% CI: 0.13 to 0.33]). The vast majority of those with stiff neck were admitted in summer (15.2%, 31/204) and 3 in winter (1.9%, 3/157). Statistical difference between season and bulging fontanelle (among <1.5 years olds) was detected with high significance (*p*<0.001) and strong correlation (*Corre* = 0.54 [95% CI=: 0.43 to 0.64]). All cases with bulging fontanelle (age 0 to 18 months [*n* = 219]) were admitted in summer (30%, 37/124). No significant differences and associations were found between the remaining demographic and clinical data.

Based on hospital records; 61.8% (220/356) of all admissions during the study period were diagnosed as having a CNS associated condition; 55.9% (199/356) as bacterial (septic) meningitis, 0.6% (2/356) brain abscess and 5.3% (19/356) febrile convulsions. The remaining 38.2% (136/356) were diagnosed as having other infections; data is provided in the Additional file 1. High significance (*p*<0.001) but weak association (*Corre* = 0.27 [95% CI: 0.19 to 0.35]) were observed between hospital diagnosis and season of admission. On cross-tabulations; amongst patients diagnosed as having BM, 50.3% (100/199) were admitted in summer and 49.8% (99/199) in winter. All patients were treated during hospitalization by first line and/or second line antibiotic therapies (Table 2).

**Table 2:** Prescriptions during hospitalization

Antibiotic Prescription During Hospitalization		Number of Cases	% Out of 354
*First Line Antibiotic Therapy (All of the 354 cases)*			
1	Ampicillin	186	52.5%
2	Penicillin	89	25.2%
3	Cephalosporin	78	22.0%
4	Quinin	1	0.3%
Antibiotic Prescription During Hospitalization		Number of Cases	% Out of 189
*Second Line Antibiotic Therapy (189 out of the 354 cases)*			
5	Ampicillin	1	1%
6	Penicillin	2	1%
7	Cephalosporin	165	87%
8	Chloramphenicol	8	4%
9	Gentamycin	4	2%
10	Quinin	9	5%

### Hospital-stay data

Period of hospital stay was evaluated; median 6 days with range of 1 to 38 days. Highly significant and strong positive association (*p*<0.001, *Corre* = + 0.77 [95% CI: 0.72 to 0.81]) were identified between the duration of hospital stay and final hospital diagnosis. Findings of cross-tabulations are summarized on Table 3.

**Table 3:** Hospital-stay data

Duration of Hospital Stay	Hospital Diagnosis	Bacterial Meningitis	Brain Abscess	Febrile Convulsions	Non CNS Infections	Number of Cases
1 to 3 Days Stay in Hospital		11/186 (5.9%)	1/2 (50%)	8/19 (42%)	94/134 (70.1%)	114
4 to 7 Days Stay in Hospital		41/186 (22%)	1/2 (50%)	9/19 (47.4%)	36/134 (26.9%)	87
8 to 14 Days Stay in Hospital		129/186 (69.4%)	–	1/19 (5.3%)	3/134 (2.2%)	133
15 to 30 Days Stay in Hospital		4/186 (2.2%)	–	1/19 (5.3%)	1/134 (0.8%)	6
More than 30 Days Stay in Hospital		1/186 (0.5%)	–	–	–	1
Number of Cases (% Out of 356)		199 ^a^ (55.9%)	2 (0.6%)	19 (5.3%)	136 ^b^ (38.2%)	

^a^Missing data for 13 cases among those with Bacterial Meningitis; the denominator for percentages among Bacterial Meningitis is 186

^b^Missing data for 2 cases among those with Non CNS Infections; the denominator for percentages among Non CNS Infections is 134

### Conventional laboratory data

All of the 503 patients were subjected to LP, nevertheless, 8.8% (44/503) of the withdrawn CSF specimens were traumatic, therefore, cytological and chemical analyses were not performed. The vast majority (94.8%, 383/404) of CSF specimens that were non-traumatic showed no WBCs (<5 cells/mm^3^). Significant WBC count (≥5 cells/mm^3^) was identified in 5.2% (21/404). Median CSF cell count for values above 5 cells/mm^3^ was 3000 with range of 50 to 33,400 cells. CSF specimens with high WBC count (>100 cells/mm^3^) showed neutrophilic predominance; reaching in average 80% and those with low WBC count (≤100 cells/mm^3^) showed lymphocytic predominance; reaching 60%. Abnormal CSF glucose concentration (< 45 or > 100 mg/dl) was seen in 11.9% (54/453). Abnormal CSF protein concentration (< 14 or > 45 mg/dl) was seen in 47.7% (215/451). Direct Gram stain preparation showed Gram positive diplococci in 0.7% (3/451 [52 missing]) and Gram negative diplococci in another 0.7%. On CSF culture for rapidly growing bacteria, all specimens that were Gram positive on direct preparations yielded *S. pneumoniae* (0.7%; *n* = 3) and all those that were Gram negative yielded *N. meningitidis* (0.7%; *n* = 3). A single bacterium was isolated from each patient; there were no polymicrobial infections. Two out of the 3 (66.7%) *N. meningitidis* isolates were identified as W135 serotype. Further laboratory data is provided in the Additional file 1.

### Findings on cases with positive bacterial aetiology

#### *Streptococcus pneumoniae*

All 3 patients with positive bacterial culture for *S. pneumoniae* presented with fever and seizures; 1/3 (33.3%) suffered also from chills, vomiting, bulging fontanel and altered mental status. She was the only female. They were all infants (1–11 months). The female and 1 of the 2 males (66.7%) were admitted in summer; the third in winter. The female CSF was bloody; the rest were turbid. Turbid specimens showed high CSF WBC count (≥1000 cells/cmm^3^) and all showed neutrophilic predominance (≥70%). All specimens had low CSF glucose level (< 45 mg/dl) and high CSF proteins level (> 45 mg/dl). All 3 patients had a confirmed hospital diagnosis of bacterial meningitis. They were all treated with penicillin, ampicillin and/or cephalosporins. Further data is provided in the Additional file 1.

#### Neisseria meningitidis

All the same, all 3 patients presented with fever and seizures. One of the 3 (33.3%); a toddler in the age group (1–5 years), suffered also from headache and vomiting. The remaining 2 (66.7%) infants (1–11 months) presented also with chills, 1 (33.3%) of them with vomiting and the other with stiff neck and bulging fontanel. *N. meningitidis* serotype W135 was identified in the isolates from the two infants. All patients were females, they all admitted in summer. Non typical conventional laboratory results were seen; the case that was presented with only 4 symptoms had a clear CSF, normal WBC count (<5 cells/mm3), normal CSF glucose level (45-100 mg/dl), and normal CSF protein level (14-45 mg/dl). The second infant had a traumatic CSF; therefore, no cytological or chemical analyses were performed. The toddler’s CSF WBC count revealed leukocytosis (> 1001 cells/mm3); low CSF glucose level (< 45 mg/dl) and high CSF protein level (> 45 mg/dl). All patients had a confirmed hospital diagnosis of septic meningitis. They were all treated with penicillin or ampicillin and cephalosporins. Further data is provided in the Additional file 1.

#### Secondary analysis using clinical prediction rules

**Fig. 2:**
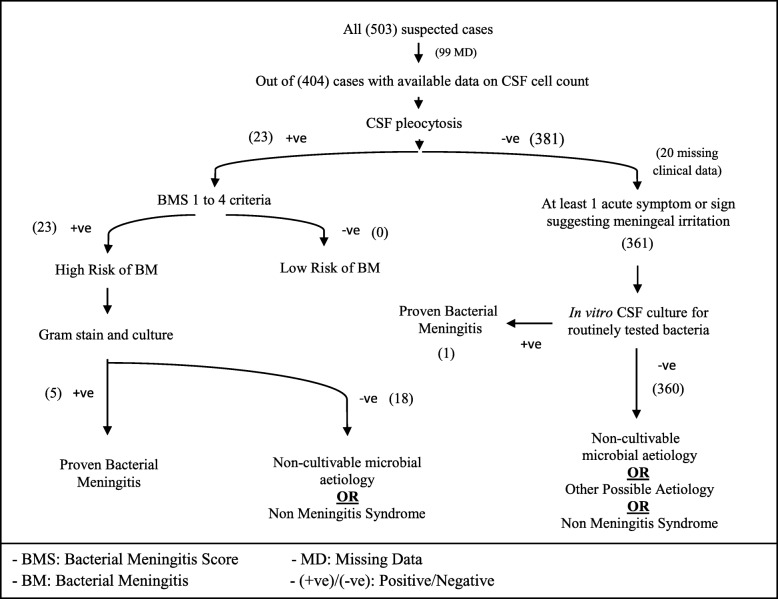
Categorization of all cases based on the Bacterial Meningitis Score and the International Classification of Diseases – Code for Bacterial Meningitis

Data on the 5th criterion (*Peripheral Blood Absolute Neutrophil Count ≥ 10,000 Cells/*μL) of the BMS was unavailable; therefore, 4 criteria were used in this analysis. When 1 or more prediction rules were fulfilled, patients were considered at *High Risk* of BM (Table 4). Patients having none of the High Risk criteria were considered at *Low Risk* of BM. All findings are described on Tables 4 and 5 and Fig. 2.

**Table 4:** Five high-risk criteria of the bacterial meningitis score

#	High-Risk criteria for bacterial meningitis	Number of cases out of 23 with Pleocytosis^a^	Number of cases out of total with available data per each variable
1	Positive CSF Gram stain	6	6 out of 451
2	CSF ANC^b^ ≥ 1000 cells/μL	19^c^	19 out of 404
3	CSF protein ≥80 mg/dl	19	25 out of 451
4	Peripheral blood ANC ≥ 10,000 cells/μL	NAD^d^	NAD
5	Presence of seizure at or before presentation	19	334 out of 361

^a^ *Pleocytosis is defined as CSF cell count > 5cells/mm* ^*3*^

^b^ *ANC: Absolute Neutrophil Count*

^c^*Count of N% from 68 to 100% in samples with CSF cell count of ≥ 1001 cells/*μL

^d^ *NAD: Data Not Available*

*The Bacterial Meningitis Score developed by Nigrovic* et al. *(2002)* [14]

**Table 5:** Re-classification according to the bacterial meningitis score and the international classification of diseases – code for bacterial meningitis

	Re-classification	Cases out of 404 ^a^		% Out of 40 ^b^
		%	Frequency	
1	Proven Infectious Meningitis	10%	40 ^b^	100%
2	Proven Bacterial Meningitis	1.5%	6	15%
3	Meningococcal meningitis	0.7%	3	7.5%
4	Pneumococcal meningitis	0.7%	3	7.5%

^a^ *Total number of febrile patients who attended the hospital during the study period and were subjected to LP were 503, however, 404 is determined as the denominator because of complete bacteriology and clinical data*

^b^ *17 cases with positive microbial origin -but with normal cellular count- along with all the 23 cases with CSF pleocytosis. Findings on microbes that are not rapid-growing-bacteria will be revealed in other publications*

## Discussion

In reference to the data from hospital records, BM was assigned for 56% of the study population. This number seems to be exaggerated since neither an *Alert* nor an *Epidemic* [5] of BM was announced by National and/or International authorities in Sudan at the time of the study. In fact, a much smaller figure of 1.5% was later identified as *Proven Bacterial Meningitis* when codes and criteria were followed (Table 5). A total of 40 cases out of the study population were found to either have *Proven Infectious Meningitis* or are *Highly Suspected* to have the disease. Accordingly the proportion of those with proven infectious meningitis among our study population would be 10% (Table 5). We found this figure to be rather diluted because of the wide denominator i.e. provisional diagnosis of infectious CNS disease was too inclusive. This shortcoming is not only limited to hospitals in Khartoum since several articles from around the world reported almost similar findings. The proportion of children who were proved to have meningitis by laboratory confirmation was always around 50% or less among those who were highly suspected and subjected to LP. Studies from the developing world reported much less estimates that are closer to ours. A study conducted in pediatric hospitals in Iran has identified meningitis in 16.8% (65/387) [19]. Countries falling in the meningitis belt reported small estimates as well. Laboratory confirmed cases among suspects during 5 years in Burkina Faso were 22% (4503/20,389) [20], 24% (279/1176) during 6 years in Ghana [21] and 26% (871/3306) in 20 years surveillance in Mali [22]. Studies in developed countries reported different results. Amarilyo et al [23] reported 54% (58/108) among meningitis suspected children. Other studies conducted by Dubos et al [24], Oostenbrink et al [25], Bonsu et al [26] and Oostenbrink et al [27] in children who underwent LP have reported comparable findings (≈ 40% prevalence of meningitis). The corresponding low value we obtained from hospital records could be attributed to lack of strict adherence to meningitis criteria, a situation that appears to be common in hospitals in developing countries. We identified only 1.5% as proven BM which was closely in agreement with published reports where only 3.7% (*n* = 122) were identified in the study of Nigrovic et al [16] among 3295 children with CSF pleocytosis. Among highly suspected cases in our study, however, a higher figure of 15% was identified as BM. This was in good agreement with previous published reports. Dubos et al [28] and Amarilyo et al [23] identified 12% (20/166) and 10.3% (6/58), respectively, as BM among suspected patients in pediatric emergency units. Similarly, Bonsu et al [26] and Oostenbrink et al [27] identified a maximum of 20%. In contrast, a national Polish survey identified bacterial aetiology in 40% (980/2475) of cases with neuroinfections in 1 year [29]; most probably a result of an outbreak in Poland.

When it comes to diagnosing a debilitating and potentially fatal illness in a pediatric population of a country located in the meningitis belt, health care officers in Sudan often tend not to dismiss a poorly suspected case. Fear of misdiagnosis or late treatment result in over diagnosis that could lead to an increased hospitalization costs as evident by the extended periods of hospitalizations (Table 3). Over diagnosis can also lead to the emergence of drug-resistant strains due to antibiotics overuse, and hence the accuracy of the national health registry becomes questionable. Therefore, the ability to monitor simple seasonal rise in disease incidence, identifying an actual epidemic and implementing the appropriate control measures will consequently be affected.

Pneumococcal and meningococcal conditions were equally identified in 7.5% among those with proven infectious meningitis in this study. A recent meta-analysis [30] covering 56 studies identified *S. pneumoniae* and *N. meningitidis* as the predominant pathogens of BM in children of all ages in all regions. In previous studies, pneumococcal disease was usually identified in relatively small number of cases. Dubos [28] reported 5% (9/166) *S. pneumoniae* meningitis among patients with CSF pleocytosis. Even though in a meningitis outbreak situation, only 6% (*n* = 149) was reported as *S. pneumoniae* among 40% with bacterial aetiology [29]. Never the less, the aforementioned meta-analysis reported *S. pneumoniae* as the most common cause of BM in children ranging from 22.5% in Europe and 41.1% in Africa [30]. A reduction in meningococcal meningitis occurrence was reported in Burkina Faso [20] and Ghana [21], where Dubos [28] and Turczyńska [29] identified *N. meningitidis* in 5% (9/166) and 9% (220/2475) respectively, closely comparable to our findings of 7.5%. On the other hand, studies in Mali reported 44% meningococcal meningitis and 31% pneumococcal meningitis [22].

All our patients were admitted in the high risky season that is described climatologically as dry compared to about 65% of all cases that were recorded in the Ghanaian study in this season [21]. This period constitutes the peak of meningococcal disease occurrence, unlike the pneumococcal occurrence that varies seasonally [31, 32]. In this study, all meningococcal patients were less than 5 years, an age group that has the highest reported incidence rate [30, 33]. Pneumococcal meningitis occurs most commonly in the very young and the very old [9], where all cases we have studied were infants aged 1 to 11 months. Fortunately, all of them have recovered and discharged despite reports on high case-fatality rates [9].

Diagnostic signs for BM in young children are unclear; they do not often exhibit the general symptoms and may only be irritable and look unwell [34]. All cases with proven BM in this study presented with high fever and seizures. Other classic symptoms, namely, neck stiffness, chills and bulging fontanelle, were seen in one third of the children only. Bulging fontanelle and admission in summer were the only parameters that showed significant statistical difference and strong association. An inflammation in the brain or the meninges can cause a bulging fontanelle [34]. Amongst the several aetiologies bacteria and viruses are the most common and these occur more frequently in summer [34]. The fact that all 37 (20%) patients who suffered from a bulging fontanelle (Table 1) presented in summer strongly suggests a microbial aetiology. However, only 2 (33.3%) of our 6 BM confirmed cases presented the symptom. In the study of Amarilyo et al [23], bulging fontanelle was present in 50% of patients with meningitis and had a positive predictive value of only 38%. Several studies have reported atypical clinical findings in young patients with BM of either meningococcal or pneumococcal aetiology [23, 35]. In fact, a guideline describing these anomalies was established [36]. Amarilyo [23] recommended that these clinical indicators should not be the sole determinants for referral to further diagnostic testing and LP.

Typical findings on CSF analysis [37, 38] were seen in all cases with *S. pneumoniae* and in only one third with *N. meningitidis*, while another third showed normal CSF picture. Normal CSF cellular count in those with positive CSF culture can be demonstrated, however, rarely [39]. Garges et al [40] concluded that BM in babies frequently occurs in the presence of normal CSF parameters, including WBC count [40].

Hib was not identified in this study probably as the result of the introduction of Hib vaccine to the pediatric population in Sudan which was first started in 1976 and later on in 2001 [13]. Following critical review of accessible publications, this finding is in conformity. The study from Burkina Faso reported *H. influenzae* in a small proportion of 2% [20] and Ghana did not identify the bacterium amongst other aetiologies of BM in children [21]. Further, Strange [41] affirmed that *H. influenzae* meningitis in children has become so rare that a case could hardly be seen. The study of Dubos [28] identified only one case with Hib meningitis (0.6%) among 166 BM suspected patients and the prospective French nationwide survey of Bingen et al [42] detected *H. influenzae* in only 2.5% (27/1084). Amongst published articles within our reach were those from Mali which stated that the country has suffered high morbidity and mortality of BM and high occurrence of *H. influenzae* meningitis [22, 43]. Hib conjugate vaccine was first introduced to children in Mali in 2005 to 2007 [43]. In 20 years surveillance (1996–2016) Mali reported *H. influenzae* in 23% [22] and a post vaccination evaluation [43] identified a decrease of 86% of Hib among high risk infants of 6 to 7 months old and an overall annual incidence drop of 74% by the second year. All the aforementioned studies, including those from Mali, and many others along with ours confirm the notion that Hib meningitis is about to be eliminated. It is worth mentioning that the Federal Ministry of Health in Sudan has been demonstrating strong commitment and good adherence to vaccination programmes provided by UNICEF and WHO [13]. The observed absence of Hib infections and the great reduction in conditions of BM caused by all other aetiologies are closely comparable to that reported by Schuchat [12] and fairly places Sudan in a better position regarding the control of such disease compared to other African countries.

## Conclusions

In conclusion, the study has indicated a significant reduction in the occurrence of BM. *S. pneumoniae* and *N. meningitidis* are the sole pathogens of pediatric BM and *H. influenzae* is no longer an aetiology. Many cases showed atypical clinical and laboratory findings. Codes and criteria for the diagnosis of BM are not followed and that consequence leads to over-diagnosis and over-prescription of antibiotics. It is therefore of paramount importance that the decision of BM should be guided by internationally validated clinical prediction rules.

## Additional file

Additional file 1: Supplementary materials are provided in the file: *BM in Children in Sudan (Supplementary Material)*. It includes 3 tables in the following order. Hospital Diagnosis (Non CNS Conditions): Shows frequencies and percentages of cases diagnosed by the hospital as having conditions affecting systems other than the CNS. Conventional Laboratory Data: Demonstrates detailed laboratory findings for all cases. Findings on Cases with Positive Bacterial Aetiology: Shows detailed findings for cases with confirmed bacterial meningitis. (DOCX 27 kb)

## Data Availability

The dataset used during the current study will be available from the corresponding author on reasonable request.

## Abbreviations

ANC: Absolute Neutrophil Count
BM: Bacterial Meningitis
BMS: Bacterial Meningitis Score
CI: Confidence Interval
CNS: Central Nervous System
Corre: Nominal by Nominal Phi and Cramer’s V Correlation
CSF: Cerebrospinal Fluid
*H. influenzae*: *Haemophilus influenzae*
Hib: *Haemophilus influenzae* type b
ICD-CMC: International Classification of Diseases – Clinical Modification Code
IQR: Inter Quartile Range
LP: Lumber Puncture
MD: Missing Data
Mdn: Median
*N. meningitidis*: *Neisseria meningitidis*
NA: Not Applied
NAD: Data Not Available
*S. pneumoniae*: *Streptococcus pneumoniae*
SD: Standard Deviation
UNICEF: United Nations International Children’s Emergency Fund
WHO: World Health Organization
