# Supplementary material for: Bacterial meningitis in Sudanese children; critical evaluation of the clinical decision using clinical prediction rules
Source: BMC Pediatr. 2019 Sep 6;19:319. doi: 10.1186/s12887-019-1684-3 (PMC6729048; doi:10.1186/s12887-019-1684-3)
Supplement: Supplementary file 1 — Supplementary materials are provided in the file: BM in Children in Sudan (Supplementary Material). It includes 3 tables in the following order. Hospital Diagnosis (Non CNS Conditions): Shows frequencies and percentages of cases diagnosed by the hospital as having conditions affecting systems other than the CNS. Conventional Laboratory Data: Demonstrates detailed laboratory findings for all cases. Findings on Cases with Positive Bacterial Aetiology: Shows detailed findings for cases with confirmed bacterial meningitis. (DOCX 27 kb) [file 12887_2019_1684_MOESM1_ESM.docx]

**Supplementary**

| Hospital Diagnosis (Non CNS* Conditions) | | | |
| --- | --- | --- | --- |
|  | **Non CNS conditions** | **Number of Cases** | **% Out of 136** |
| **1** | Tonsillitis | 40 | 29.4% |
| **2** | Bronchitis | 1 | 0.7% |
| **3** | Pneumonia | 66 | 48.5% |
| **4** | Sepsis | 5 | 3.7% |
| **5** | Malaria | 13 | 9.6% |
| **6** | Urinary Tract Infection | 1 | 0.7% |
| **7** | Poliomyelitis | 1 | 0.7% |
| **8** | Fever of Unknown Origin | 2 | 1.5% |
| ***Combined Infections*** | | | |
| **9** | Tonsillitis & Severe Pneumonia | 4 | 2.9% |
| **10** | Severe Malaria & Pneumonia | 3 | 2.2% |
| **CNS: Central Nervous System* | | | |

| Conventional Laboratory Data | | | | | | |
| --- | --- | --- | --- | --- | --- | --- |
| **1** | **CSF Cell Count** | **Frequency** | | **% Out of 404** | | **% Out of CSF with Abnormal Finding (*n=*21)** |
|  | < 5 cells/mm3 | 383 | | 94.8% | | NA |
|  | 21 to 100 cells/mm^3^ | 2 | | 0.5% | | 9.5% |
|  | 101 to 1000 cells/mm^3^ | 4 | | 1% | | 19.1% |
|  | ≥1001 cells/mm^3^ | 15 | | 3.7% | | 71.4% |
| **2** | **CSF Glucose Concentration** | **Mean + SD** | **Range** | **Frequency** | **% Out of 453** | **% Out of CSF with Abnormal Finding (*n=*54)** |
|  | Normal (45-100mg/dl) | 70 + 13.4 | 45 to 100 mg/dl | 399 | 88.1% | NA |
|  | High (> 100mg/dl) | 141 + 39 | 101 to 221 mg/dl | 20 | 4.4% | 37% |
|  | Low (< 45mg/dl) | 26 + 14 | 0 to 44 mg/dl | 34 | 7.5% | 63% |
| **3** | **CSF Protein Concentration** | **Mean + SD** | **Range** | **Frequency** | **% Out of 451** | **% Out of CSF with Abnormal Finding (*n=*215)** |
|  | Normal (14-45mg/dl) | 24 + 7 | 14 to 45mg/dl | 236 | 52.3% | NA |
|  | High (> 45mg/dl) | Mdn 67mg/dl | 46 to 1,072 mg/dl | 56 | 12.4% | 26% |
|  | Low (< 14mg/dl) | 10 + 3 | 1.7 to 13.5 mg/dl | 159 | 35.3% | 74% |
| **4** | **Bacterial Growth** | **Gram Stain** | | **Frequency** | **% Out of 503** | **% Out of CSF with Abnormal Finding (*n=*6)** |
|  | No Bacterial Growth | No bacteria seen | | 497 | 98.6% | NA |
|  | *Neisseria meningitidis* | Gram Negative Diplococci | | 3 | 0.7% | 50% |
|  | *Streptococcus pneumoniae* | Gram Positive Diplococci | | 3 | 0.7% | 50% |
| **5** | **Macroscopical Examination** | **Frequency** | | | **% Out of 503** | |
|  | Clear | 428 | | | 85.4% | |
|  | Turbid | 13 | | | 2.6% | |
|  | Bloody | 16 | | | 3.2% | |
|  | Traumatic | 44 | | | 8.8% | |
| *NA: Not Applied*  *SD: Standard Deviation*  *Mdn: Median* | | | | | | |

| Findings on Cases with Positive Bacterial Aetiology | | | | | |
| --- | --- | --- | --- | --- | --- |
| ***Streptococcus pneumoniae*** | | | | | |
|  | **Parameters** | | **Patient 1** | **Patient 2** | **Patient 3** |
| **1** | Sex | | Female | Male | Male |
| **2** | Age | | 3 Months | 6 Months | 5 Months |
| **3** | Age in Groups | | Infant (1-11 months) | Infant (1-11 months) | Infant (1-11 months) |
| **4** | Season of Admission | | Summer | Summer | Winter |
| **5** | Presentation with Fever and Seizures | | Yes | Yes | Yes |
| **6** | Presentation with other Symptoms of Meningeal Irritation | | Chills, Vomiting, Bulging Fontanelle, Altered Mental Status | Vomiting | No |
| **7** | CSF Macroscopical Examination | | Bloody | Turbid | Turbid |
| **8** | CSF Microscopical Examination | | Gram positive diplococci | Gram positive diplococci | Gram positive diplococci |
| **9** | CSF WBC Count | | NA | Leukocytosis (340 cells/mm^3^) | Leukocytosis (26000 cells/mm^3^) |
| **10** | CSF Absolute Neutrophil Count | | NA | 84% | 70% |
| **11** | CSF Glucose Level | | Low (16 mg/dl) | Low (8 mg/dl) | Low (29 mg/dl) |
| **12** | CSF Proteins Level | | High (415 mg/dl) | High (333 mg/dl) | High (499 mg/dl) |
| **13** | Hospital Diagnosis | | Bacterial Meningitis | Bacterial Meningitis | Bacterial Meningitis |
| **14** | Antibiotic Prescriptions | | Ampicillin | Penicillin/Cephalosporin | Ampicillin/Samixon |
| ***Neisseria meningitidis*** | | | | | |
|  | **Parameters** | **Patient 1** | | **Patient 2** | **Patient 3** |
| **1** | Sex | Female | | Female | Female |
| **2** | Age | 2.25 Years | | 11 months | 2 months |
| **3** | Age in Groups | Toddler (1-5 years) | | Infant (1-11 months) | Infant (1-11 months) |
| **4** | Season of Admission | Summer | | Summer | Summer |
| **5** | Presentation with Fever and Seizures | Yes | | Yes | Yes |
| **6** | Presentation with other Symptoms of Meningeal Irritation | Headache, Vomiting | | Chills, Vomiting | Chills, Stiff Neck, Bulging Fontanelle |
| **7** | CSF Macroscopical Examination | Bloody | | Clear | Traumatic |
| **8** | CSF Microscopical Examination | Gram negative diplococci | | Gram negative diplococci | Gram negative diplococci |
| **9** | CSF WBC Count | Leukocytosis (3000 cells/mm^3^) | | Normal (˂5 cells/mm^3^) | NA |
| **10** | CSF Glucose Level | Low (18 mg/dl) | | Normal (90 mg/dl) | NA |
| **11** | CSF Proteins Level | High (259 mg/dl) | | Normal (20 mg/dl) | NA |
| **12** | *N. meningitidis* Serotype W135 | Not Identified | | Identified | Identified |
| **13** | Hospital Diagnosis | Bacterial Meningitis | | Bacterial Meningitis | Bacterial Meningitis |
| **14** | Antibiotic Prescriptions | Ampicillin/Cephalosporin | | Penicillin/Cephalosporin | Penicillin/Cephalosporin |
|  | *NA: Not Applied* | | | | |
